# Posterolateral ankle ligament injuries affect ankle stability: a finite element study

**DOI:** 10.1186/s12891-016-0954-6

**Published:** 2016-02-24

**Authors:** Zhao-Jin Zhu, Yuan Zhu, Jing-Feng Liu, Yong-Ping Wang, Gang Chen, Xiang-Yang Xu

**Affiliations:** Orthopedics Department 3, Ruijin Hospital Affiliated to Shanghai Jiao Tong University School of Medicine, Shanghai, 200025 China

**Keywords:** Posterolateral ankle ligaments, Posterolateral ankle instability, Finite element (FE), PTFL, CFL, PITFL

## Abstract

**Background:**

We have already discovered 23 patients during the work of the outpatient department and operations whose unstable signs on the posterolateral ankle. The anterior drawer test demonstrated normal during the physical examinations while the spaces of the posterior tibiotalar joints increased in stress X-ray plain films. ATFL intact and posterolateral ligaments lax were found during operations too. It is important to make existence claims and illuminate the mechanism of posterolateral ankle instability.

**Methods:**

A finite element model of the ankle was established for simulating to cut off posterolateral ligaments in turn. Ankle movements with tibia rotation under load on five forefoot positions were simulated as well.

**Results:**

The difference values with tibia external rotation were negative, and the positive results occurred with tibia internal rotation. The tibia-talus difference values in some forefoot positions were 2 ~ 3 mm after PTFL together with CFL or/and PITFL were cut off. The tibula-talus difference values were 2.21 ~ 2.76 mm after both PTFL and CFL were cut off. The tibia-fibula difference values were small. The difference values increased by 2 ~ 5 mm after cutting off the PITFL.

**Conclusions:**

Posterolateral ankle ligaments, especially CFL and PITFL, play a significant role in maintaining ankle stability. The serious injuries of both CFL and PITFL would affect posterolateral ankle stabilities. PITFL was important to subtalar joint stability.

## Background

The ankle joint is formed by the articulation of the lower leg bones tibia and fibula with the talus, including subtalar (talus-calcaneus) joint in the broad sense. The ankle connects the foot with the leg. Ankle sprains with ligament injuries often occur among athletes when landing to uneven surfaces, with sudden sideways or twisting movements of forefoot. Even treated appropriately, acute sprains often turn into chronic ankle instabilities (CAI) after recurrent sprains last for more than half a year. CAI includes a cluster of chronic symptoms characterized by recurrent sprains and ‘giving way’ feeling [1, 2], comprising with obstinate joint pains or osteoarthritis, and often needs to perform surgery of arthroscopic debridement, ligament repairs or reconstructions, even ankle fusion or total ankle replacement [3–5].

It’s important to keep dynamic and static ankle stability and health intact ligaments for support and movement functions of foot and ankle. As we know, injuries of the ligaments such as anterior talofibular ligament (ATFL), calcaneofibular ligament (CFL) and deltoid ligament lead to ankle instability [6–21]. But no attentions have been given to the relationship between posterolateral ankle ligaments and ankle instability.

The biomechanics is difficult to carry on within foot and ankle surgery research for lack of ankle cadavers, though developed well in sports medicine. Finite element analysis (FEA), benefited from the development of computer technology, is very effective for foot and ankle biomechanics research.

FEA simulates actual ankle systems with minimum errors by mathematics approximation methods. A large number of literature about acute ankle sprains, CAI, ligament repairs or reconstructions, and joint replacements in the field of sports medicine can be obtained easily [22–26]. It’s difficult to develop an appropriate animal model to simulate the human ankle joint, so FEA is naturally appropriate for human ankle research for the properties of high effect, arbitrary point analyzable, and consistent results.

More than 2500 outpatients suffered from CAI have been diagnosed and 250 of 5000 surgical patients had CAI symptoms in foot and ankle center of Shanghai Ruijin Hospital in recent 5 years. We have already discovered 23 patients during the work of the outpatient department and operations whose unstable signs on the posterolateral ankle. The anterior drawer test demonstrated normal during the physical examinations while the spaces of the posterior tibiotalar joints increased in stress X-ray plain films. ATFL intact and posterolateral ligaments lax were found during operations too. It is important to make existence claims and illuminate the mechanism of posterolateral ankle instability. Posterolateral ankle ligaments may provide support to ankle torsion and inversion stabilities according their anatomic features [27, 28]. We developed an ankle three-dimensional finite element model to simulate the ankle stability changes after posterolateral ligament damages. The purpose is to evaluate the influence of posterolateral ankle ligament injuries on ankle instability.

## Methods

### Model development

First of all, we have a healthy male adult volunteer to do a 64-row spiral CT scanning of right foot, ankle and lower leg at 2 mm intervals from the coronal plane with forefoot unload neutral position, who was 47 years old, with a height of 171 cm and a weight of 60 kg, free from ankle joint diseases. The scanning was approved by the Ethics Committee of Ruijin Hospital Affiliated to Shanghai Jiao Tong University School of Medicine and we compiled with the Helsinki Declaration and the principle of informed consent. The volunteer agreed to participate the test verbally, regarding it as a health examination. Then we exported the DICOM formation data of the CT images to a compact disc from the computer of Medical Imaging Center.

The images were distinguished in MIMICS v17.0 (Materialise, Leuven, Belgium) to be divided into small regions that fit with triangles to reconstruct the bone geometry. The reverse engineering software Geomagic Studio software v12.0 (Geomagic Inc., Research Triangle Park, NC) reduced noise levels of the STL formation point cloud data got from MIMICS software and made smooth polygons of bone surface. We imported the created non-uniform rational B-spline (NURBS) curve model into pre-processing finite element software Hypermesh 13.0 to finish reassembly. The mesh density was set after a convergence study. Firstly, we built the bone surface with triangle elements. Then we built cortical bones with 1 mm bias units and Under them cancellous bones with common nodes of tetrahedral elements. Joints were simulated by bone surface bias elements. Tendons and ligaments attach to bones with truss units as an actual anatomic relationship. Joint contact effects were simulated by setting hard contact between both joint bones. A total of 23,047 elements and 6,821 nodes were used for the bone, cartilage and ligament establishments (Table 1).

**Table 1:** The modulus of elasticity, Poisson’s ratio, number of elements, and nodes for each of the material in the finite element foot and ankle model

Material	Young’s Modulus/Mpa	Poisson’s ratio	Elements	Nodes
Bone	7300	0.3	20615	5019
Ligament	260	0.49	36	72
Cartilage	12	0.42	2396	1730

**Fig. 1:**
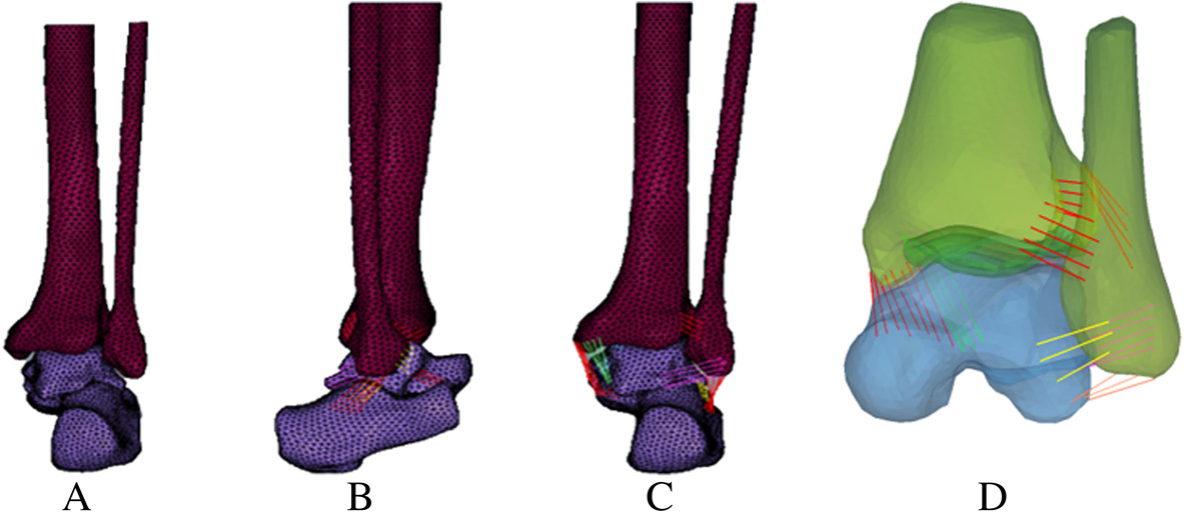
Finite element model of ankle joint (**a**), lateral view (**b**) and posterior view (**c**), and assembly of ligaments and articular cartilage (**d**)

The fixator model was analyzed in ABAQUS finite element software v6.9 (ABAQUS Inc., Pawtucket, RI). Different stages of model assembly are shown in Fig. 1.

**Fig. 2:**
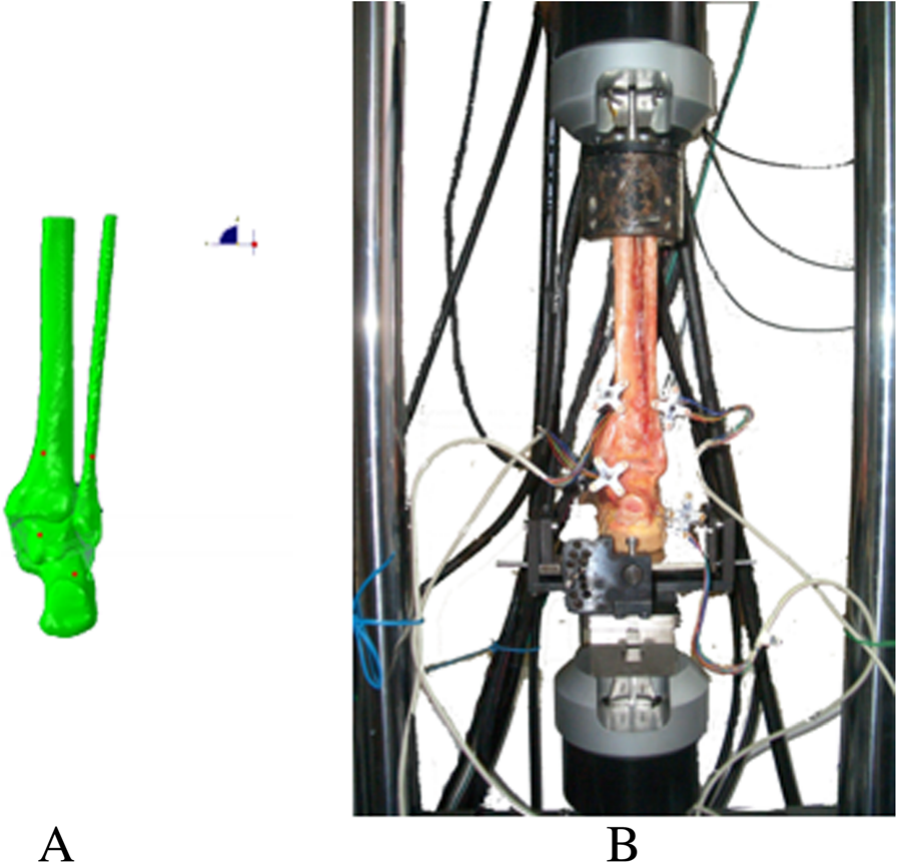
Four rigid marker points in FEA (**a**) and biomechanical experiment (**b**)

In biomechanical experiment and FEA, we regarded the bone as the rigid body. In an equivalent rigid body system, the results can be analyzed by relatively simple standard procedures. The position vector of arbitrary elements in a rigid body isn’t equal to each other, but displacement, velocity and acceleration are constant. In biomechanical experiments, motions of a rigid body are represented by motions of the marker point on it, wherever the marker is. The rigid body movement is modeled as the marker movement in experiments. Four rigid markers in FEA and biomechanical experiment are shown in Fig. 2.

### Material properties

CT images provide us higher quality bone image data to reconstruct the rigid body of the biomechanical experiment, while sacrifice the ligament image quality. So ligaments were indicated as linear elastic modulus by the line segments between origin and insert for primary study. The bone base and the articulating surface of the ankle were meshed as a rigid body and a rigid surface, respectively. The properties (Young’s modulus, Poisson’s ratio) of the bone, ligament and cartilage were assigned according to the previous literature and are shown in Table 1 [29–31].

### Loading and boundary conditions

A reference point was set at the top of the tibia, and the coupling relationship of the upper end of tibia and fibula was established. Ligaments were represented by 2-node truss units simulating the non-compression characteristics which can only bear traction powers. Ligament functions were simulated with coupling units and changeable vector loads. Ankle surface contact was simulated with the face-to-face nonlinear universal interaction. Articular cartilages were set by 2 mm offset tibia and talus contact elements. Ankle surface contacts abided tangential Coulomb friction and the friction coefficient was 0.1. The hard contacts in vertical direction were the nonlinear penalty function.

To simulate the biomechanical test condition of the body weight load of the human ankle and to improve the convergence of FEA model, the loads were undertaken with three steps. Firstly, the upper of the tibia was applied a load of 58.8 N to establish a stable relationship between the contact joint bones. Then, the joint surface beard a vertical 588 N body weight through the tibia-fibula combination to get a maximum contact in order to form a stable ankle bearing relationship. Finally, the tibia-fibula was applied a torque of 10 N.m for internal and external rotation.

**Fig. 3:**
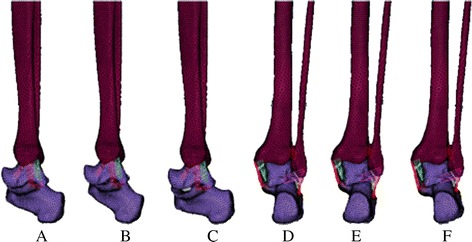
Five positions in FEA. **a** and **d**. neutral; **b**. plantar flexion; **c**. dorsiflexion; **e**. inversion; **f**. eversion

After material properties and boundary conditions were properly setup in the foot and ankle finite element model, ligaments cutting off and three-dimension analysis were performed. Posterior talus-fibular ligament (PTFL), CFL and posterior inferior tibiofibular ligament (PITFL) were cut off in turn and each step got three-dimensional data of marker points in forefoot neutral, 10° plantar flexion, 10° dorsiflexion, 10° inversion, and 10° eversion positions (Fig. 3). The system used microcomputer to deal with space data, and derives three-dimensional spatial information of marker points.

### Model validation

In the finite element simulation, the four markers, loader and rotation, and forefoot positions were same as the biomechanical experiment (Fig. 6). The ligaments were set according their anatomy origins and inserts [26, 32, 33]. The promoted finite element model was validated by comparing simulation results with biomechanical experiment results in the same conditions, the agreement is obtained after analyzing different interventions, and the FEA results have a high credibility.

### Evaluation index

**Fig. 4:**
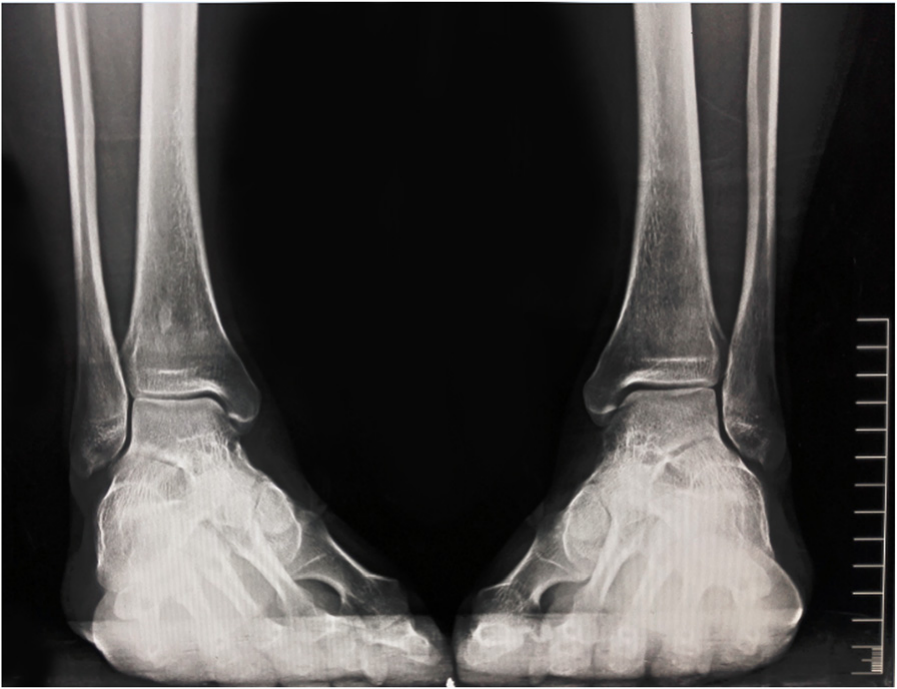
Ankle mortise view x-ray photography. The lucent area of tibia-talus or fibula-talus joint is about 4 mm, including a small amount of synovial fluid and 2 mm + 2 mm non-visualized articular cartilage

The thickness of ankle lucent area in an ankle mortise view x-ray photography is about 4 mm, including a small amount of synovial fluid and 2 mm + 2 mm non-visualized articular cartilage of tibia-talus or fibula-talus joint surfaces. The two ankle cartilage surfaces contact closely and the normal space distance between them can be even neglected, and the distance shifts > =2 mm than normal were identified as instability (Fig. 4). The difference value was calculated by subtracting the original distance from the post-cut distance. After reviewing the literature and consulting ankle surgery professor Xiangyang Xu of Shanghai Ruijin Hospital in China and professor Beet Hintermann of Kantonsspital in Switzerland, the two are both famous international experts in the foot and ankle surgery, we took the relative distance change of the two rigid markers > =2 mm as a positive result [34–38].

### Data analysis

The values of finite element analysis are exact, unique. All statistical analyses were performed in GraphPad Prism v6.05 for Windows (GraphPad Software, Inc., La Jolla, CA, USA) and Excel in Microsoft Office 2016 (Microsoft Corporation, Redmond, USA). We had the corresponding difference values between post-cut and normal distances of each two markers in Excel and converted them into histograms by GraphPad Prism software.

## Results

### The corresponding difference values between post-cut and normal tibia-talus distances (Fig. 5)

The difference values of all the five forefoot positions with tibia external rotation were no more than 1 mm. The value of forefoot eversion with tibia internal rotation after cutting off both the PTFL and CFL was 2 mm. After cutting off all the three ligaments, the values of forefoot dorsiflexion, plantar flexion, inversion and eversion with tibia internal rotation were 2 mm,−3 mm,−2 mm and 3 mm, respectively. These results were identified as positive.

**Fig. 5:**
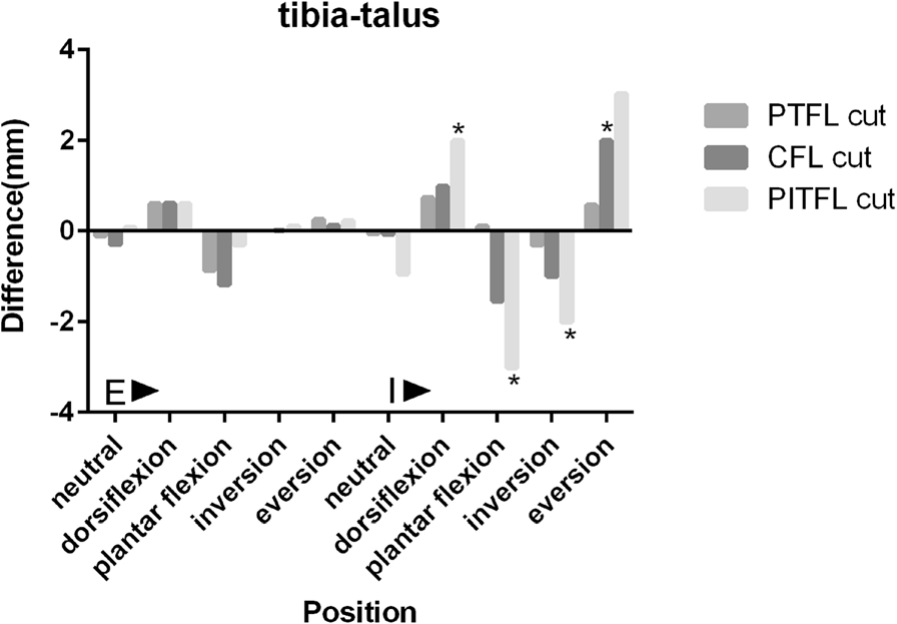
The corresponding difference values between post-cut and normal tibia-talus distances in five forefoot positions with tibia external and internal rotations. *indicated positive results. E: Tibia external rotation with a 10 N.m torque and a 588 N vertical load; I: Tibia internal rotation with a 10 N.m torque and a 588 N vertical load

### The corresponding difference values between post-cut and normal fibula-talus distances (Fig. 6)

The difference values of all the five forefoot positions with tibia external rotation were no more than 1 mm. The difference values of forefoot plantar flexion, inversion and eversion with tibia internal rotation increased by 2.21 mm, 2.76 mm and 2.29 mm after cutting off both the PTFL and CFL. All these results were identified as positive.

**Fig. 6:**
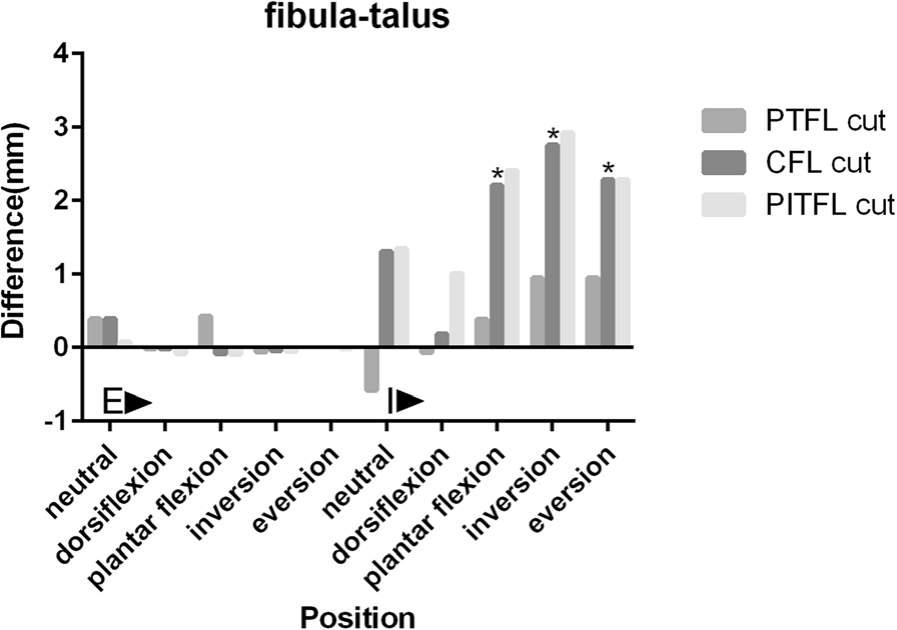
The corresponding difference values between post-cut and normal fibula-talus distances in five forefoot positions with tibia external and internal rotations. *indicated positive results. E: Tibia external rotation with a 10 N.m torque and a 588 N vertical load; I: Tibia internal rotation with a 10 N.m torque and a 588 N vertical load

### The corresponding difference values between post-cut and normal tibia-fibula distances (Fig. 7)

The difference values of all the five forefront positions were smaller than 2 mm and all of these were identified as negative consequences. The difference values of all positions with tibia external rotation of were smaller than 0.3 mm, and the biggest change of the difference value among the five positions with tibia internal rotation was only about 1 mm. These results showed that the posterolateral ligaments are not important to tibia-fibula stability.

**Fig. 7:**
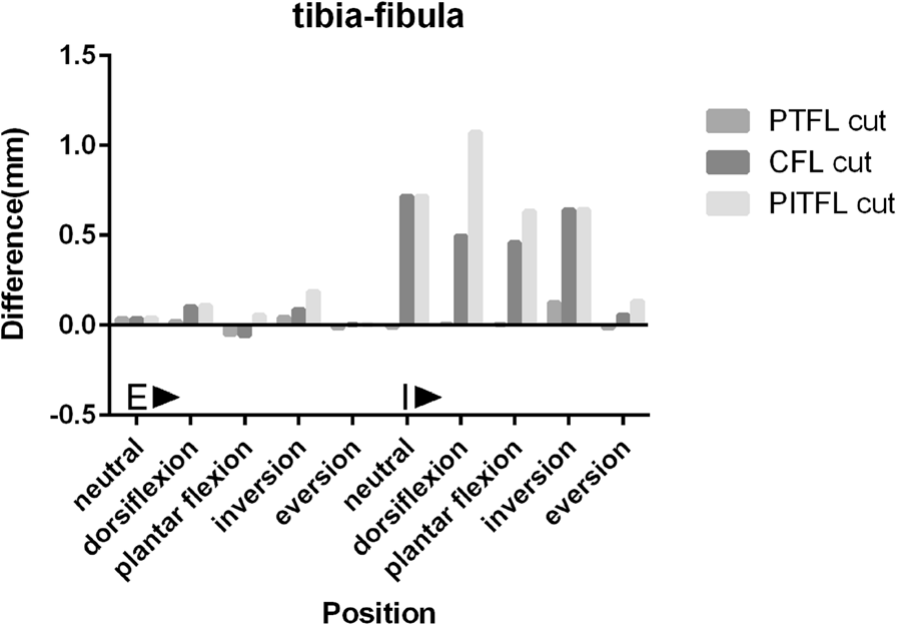
The corresponding difference values between post-cut and normal tibia-fibula distances in five forefoot positions with tibia external and internal rotations. E: Tibia external rotation with a 10 N.m torque and a 588 N vertical load; I: Tibia internal rotation with a 10 N.m torque and a 588 N vertical load

### The corresponding difference values between post-cut and normal talus-calcaneus distances (Fig. 8)

The difference values of all the five forefoot positions with tibia external and internal rotation increased by 2 ~ 5 mm after PITFL cut off at last. And obviously, these results were identified as positive. However, the difference values smaller than 0.3 mm after we cut off both the PTFL and CFL. So not CFL or PTFL but PITFL was important for maintaining subtalar joint stability.

**Fig. 8:**
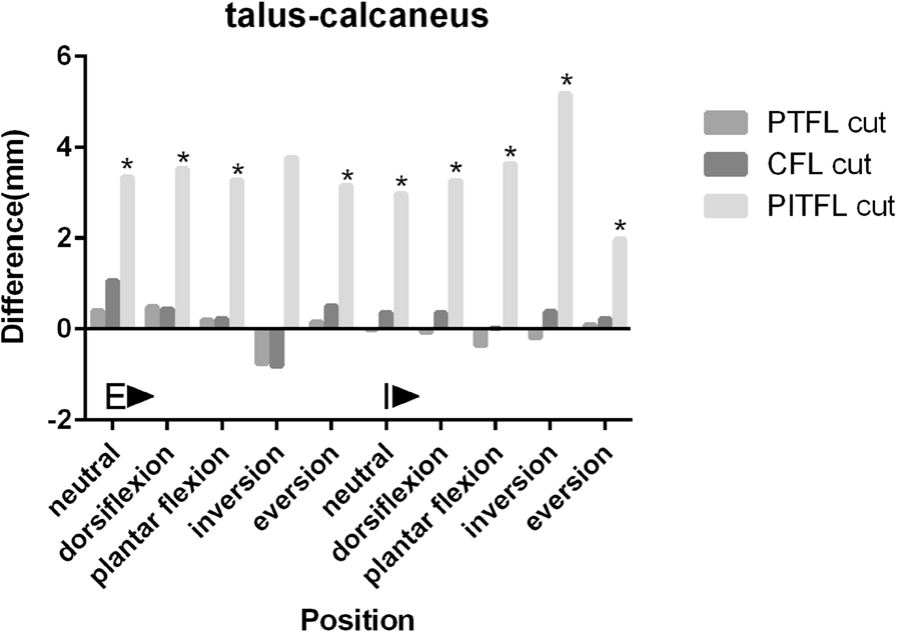
The corresponding difference values between post-cut and normal talus-calcaneus distances in five forefoot positions with tibia external and internal rotations. *indicated positive results. E: Tibia external rotation with a 10 N.m torque and a 588 N vertical load; I: Tibia internal rotation with a 10 N.m torque and a 588 N vertical load

## Discussion

Ankle injuries are the most common accounting for 14 % ~ 23 % percent of all sporting injuries. The biomechanical mechanism is very important for the research of ankle joint diseases but the specimens required in the experiments are not easy to obtain. As we know, the finite element has already been a very effective tool to simulate many biomechanics experiments including orthopedics tests since Brekelmans et al. first introduced FEA to the field of orthopaedics with femur in 1972 [39].

The three-dimensional finite element models for ankle fractures, anterior lateral ligament injury, and total ankle replacement have already been widely reported. However, the ankle finite element model of the posterior lateral ligament injuries hasn’t been studied so far. We established a three-dimensional finite element model of the ankle to test whether exist a posterolateral ankle instability, in that bones were deemed to be nonlinear elastic materials and ligaments to be linear materials for primary study. The ligaments were simulated with line segments because they were underdeveloped in CT images. The consequences proclaimed that the ankle instability can occur when CFL and PITFL were cut off.

Our results show that posterolateral ligaments mainly contribute to hold the ankle stability with tibia internal rotation and they are not beneficial to the ankle stability with tibia external rotation. The corresponding difference values between post-cut and normal distances were smaller than 2 mm with tibia external rotation. With the posterolateral ligaments were cut off one by one, the ankle instability degrees increased. PTFL cut off alone didn’t lead to an ankle instability. CFL cut off afterwards resulted in the ankle instability in some forefoot positions. Acting with the ankle instability, soft tissues such as ligaments, articular capsule and peripheral nerves around the joint would be hurt badly and the osteochondral lesions of the talus will occur. At last, after cutting off PITFL, tibia-talus difference values of forefoot dorsiflexion and eversion and fibula-talus difference value of forefoot eversion had the increases of more than 2 mm, which would aggravate damages of ankle soft tissues around and become a vicious circle. On the other hand, with forefoot plantar flexion and eversion, the decreases of tibia-talus difference values and the increases of fibula-talus difference values were more than 2 mm, then the lateral instability and medial impingement coexisted, and a shear force would result in the cartilage wear and tear in the tibia side and ligaments and nerve injuries in the fibular side, more often than not an operation is necessary in the end. We consider the posterolateral ankle instability caused by posterolateral ligament injuries may be an objective disorder and we have dealt with 23 such outpatients till now in Shanghai Ruijin hospital. Clanton et al. evaluated allograft reconstruction of ATFL alone and they wish to assess the effects of CFL reconstruction in further research [40]. Our study proclaimed that the injuries of CFL together with PITFL will cause the posterolateral ankle instability. We have just finished another experiment to assess the role of CFL to the ankle instability and the effect of CFL reconstruction.

The talus-calcaneus difference values increased obviously for 2 ~ 5 mm after cutting the PITFL off and this would result in the subtalar joint instability, while the tibia-fibula difference values shifted smaller, what’s the mechanism? Posterolateral ankle ligament injuries may lead to significant subtalar joint instability. We will try to shed light on the importance of PITFL to posterolateral ankle and subtalar stabilities in the future.

We haven’t found reports of the posterolateral ankle instability so far. Our study by biomechanical experiment and FEA model shows that CFL together with PITFL cut off can result in a posterolateral ankle instability. Talus-calcaneus difference values shifted obviously after PITFL was cut off. We got credible preliminary FEA results now. No matter which kind of chronic soft tissue injury diseases we faced, we can always cure them by restoring the dynamic balance. Our results provide a valuable theoretical base for further study with roles, mechanisms and treatment strategies of posterolateral ankle ligament injuries. We have not got the overall clinical epidemiological data of posterolateral ankle ligaments injuries at the initial stage of the research, let alone diagnoses and treatments, which are the problems have to be solved in the future. In the other hand, how about the results of cutting off PITFL or CFL first? Our purpose was to clarity the existence of the ankle instability incurred by posterolateral ankle ligaments injuries, and we got it in the end. Cutting the ligaments by different turns is a problem need to be study in the future.

## Conclusions

The difference values with tibia external rotation were negative, and the positive results occurred with tibia internal rotation. The tibia-talus difference values in some forefoot positions were 2 ~ 3 mm after PTFL together with CFL or/and PITFL were cut off. The tibula-talus difference values were 2.21 ~ 2.76 mm after both PTFL and CFL were cut off. The tibia-fibula difference values were small. The difference values increased by 2 ~ 5 mm after cutting off the PITFL.

Posterolateral ankle ligaments, especially CFL and PITFL, play a significant role in maintaining ankle stability. The serious injuries of both CFL and PITFL would affect posterolateral ankle stabilities. PITFL was important to subtalar joint stability.

## Abbreviations

ATFL: anterior talofibular ligament
CAI: chronic ankle instability
CFL: calcaneofibular ligament
FEA: finite element analysis
PITFL: posterior inferior tibiofibular ligament
PTFL: posterior talofibular ligament

## References

[1] Rios JL, Gorges AL, Dos Santos MJ (2015). Individuals with chronic ankle instability compensate for their ankle deficits using proximal musculature to maintain reduced postural sway while kicking a ball. Hum Mov Sci.

[2] Freeman MA (1965). Instability of the foot after injuries to the lateral ligament of the ankle. J Bone Joint Surg (Br).

[3] Webster KA, Gribble PA (2013). A comparison of electromyography of gluteus medius and maximus in subjects with and without chronic ankle instability during two functional exercises. Physical therapy in sport: official journal of the Association of Chartered Physiotherapists in Sports Medicine.

[4] Wikstrom EA, Hubbard TJ (2010). Talar positional fault in persons with chronic ankle instability. Arch Phys Med Rehabil.

[5] Younger AS, Wing KJ, Glazebrook M, Daniels TR, Dryden PJ, Lalonde KA, Wong H, Qian H, Penner M (2015). Patient expectation and satisfaction as measures of operative outcome in end-stage ankle arthritis: a prospective cohort study of total ankle replacement versus ankle fusion. Foot & ankle international.

[6] Yong R, Lai KW, Ooi LH (2015). Ankle lateral ligament reconstruction for chronic instability. J Orthop Surg (Hong Kong).

[7] Miyamoto W, Takao M, Yamada K, Matsushita T (2014). Accelerated Versus Traditional Rehabilitation After Anterior Talofibular Ligament Reconstruction for Chronic Lateral Instability of the Ankle in Athletes. Am J Sports Med.

[8] Boukhris J, Mojib R, Mezghani S, Jaeger JH (2014). Castaing procedure in the surgical treatment of chronic lateral ankle instability (about a series of 52 cases). Pan Afr Med J.

[9] Usuelli FG, Mason L, Grassi M, Maccario C, Ballal M, Molloy A (2014). Lateral ankle and hindfoot instability: a new clinical based classification. Foot Ankle Surg.

[10] Prissel MA, Roukis TS (2014). All-inside, anatomical lateral ankle stabilization for revision and complex primary lateral ankle stabilization: a technique guide. Foot Ankle Spec.

[11] Matsui K, Takao M, Miyamoto W, Innami K, Matsushita T (2014). Arthroscopic Brostrom repair with Gould augmentation via an accessory anterolateral port for lateral instability of the ankle. Arch Orthop Trauma Surg.

[12] Kovaleski JE, Heitman RJ, Gurchiek LR, Hollis JM, Liu W, Pearsall AW (2014). Joint stability characteristics of the ankle complex after lateral ligamentous injury, part I: a laboratory comparison using arthrometric measurement. J Athl Train.

[13] Wang H, Gu Z, Liu Y, Xu J, Jan J, Zhang J, Peng C (2015). Effectiveness of Surgery in Treatment of Ankle Fractures Associated with Deltoid Ligament Injury. Zhongguo Xiu Fu Chong Jian Wai Ke Za Zhi.

[14] Clanton TO, Williams BT, James EW, Campbell KJ, Rasmussen MT, Haytmanek CT, Wijdicks CA, LaPrade RF (2015). Radiographic Identification of the Deltoid Ligament Complex of the Medial Ankle. Am J Sports Med.

[15] Chun KY, Choi YS, Lee SH, Kim JS, Young KW, Jeong MS, Kim DJ (2015). Deltoid Ligament and Tibiofibular Syndesmosis Injury in Chronic Lateral Ankle Instability: Magnetic Resonance Imaging Evaluation at 3 T and Comparison with Arthroscopy. Korean J Radiol.

[16] Ziai P, Benca E, Skrbensky GV, Wenzel F, Auffarth A, Krpo S, Windhager R, Buchhorn T (2015). The role of the medial ligaments in lateral stabilization of the ankle joint: an in vitro study. Knee surgery, sports traumatology, arthroscopy: official journal of the ESSKA.

[17] Campbell KJ, Michalski MP, Wilson KJ, Goldsmith MT, Wijdicks CA, LaPrade RF, Clanton TO (2014). The ligament anatomy of the deltoid complex of the ankle: a qualitative and quantitative anatomical study. The Journal of bone and joint surgery American volume.

[18] Savage-Elliott I, Murawski CD, Smyth NA, Golano P, Kennedy JG (2013). The deltoid ligament: an in-depth review of anatomy, function, and treatment strategies. Knee surgery, sports traumatology, arthroscopy: official journal of the ESSKA.

[19] Crim JR, Beals TC, Nickisch F, Schannen A, Saltzman CL (2011). Deltoid ligament abnormalities in chronic lateral ankle instability. Foot & ankle international.

[20] Watanabe K, Kitaoka HB, Berglund LJ, Zhao KD, Kaufman KR, An KN (2012). The role of ankle ligaments and articular geometry in stabilizing the ankle. Clin Biomech (Bristol, Avon).

[21] Rammelt S, Schneiders W, Grass R, Rein S, Zwipp H (2011). Ligamentous injuries to the ankle joint. Z Orthop Unfall.

[22] Syngellakis S, Arnold MA, Rassoulian H (2000). Assessment of the non-linear behaviour of plastic ankle foot orthoses by the finite element method. Proc Inst Mech Eng H J Eng Med.

[23] Xu C, Zhang MY, Lei GH, Zhang C, Gao SG, Ting W, Li KH (2012). Biomechanical evaluation of tenodesis reconstruction in ankle with deltoid ligament deficiency: a finite element analysis. Knee surgery, sports traumatology, arthroscopy : official journal of the ESSKA.

[24] Terrier A, Larrea X, Guerdat J, Crevoisier X (2014). Development and experimental validation of a finite element model of total ankle replacement. J Biomech.

[25] Reggiani B, Leardini A, Corazza F, Taylor M (2006). Finite element analysis of a total ankle replacement during the stance phase of gait. J Biomech.

[26] Anderson DD, Goldsworthy JK, Li W, James Rudert M, Tochigi Y, Brown TD (2007). Physical validation of a patient-specific contact finite element model of the ankle. J Biomech.

[27] Raasch WG, Larkin JJ, Draganich LF (1992). Assessment of the posterior malleolus as a restraint to posterior subluxation of the ankle. The Journal of bone and joint surgery American volume.

[28] Rasmussen O (1985). Stability of the ankle joint. Analysis of the function and traumatology of the ankle ligaments. Acta Orthop Scand Suppl.

[29] Gefen A, Megido-Ravid M, Itzchak Y, Arcan M (2000). Biomechanical analysis of the three-dimensional foot structure during gait: a basic tool for clinical applications. J Biomech Eng.

[30] Siegler S, Block J, Schneck CD (1988). The mechanical characteristics of the collateral ligaments of the human ankle joint. Foot Ankle.

[31] Anderson DD, Goldsworthy JK, Shivanna K, Grosland NM, Pedersen DR, Thomas TP, Tochigi Y, Marsh JL, Brown TD (2006). Intra-articular contact stress distributions at the ankle throughout stance phase-patient-specific finite element analysis as a metric of degeneration propensity. Biomech Model Mechanobiol.

[32] Hartford JM, Gorczyca JT, McNamara JL, Mayor MB (1995). Tibiotalar contact area. Contribution of posterior malleolus and deltoid ligament. Clin Orthop Relat Res.

[33] Liu Q, Zhang K, Zhuang Y, Li Z, Yu B, Pei G (2013). Analysis of the stress and displacement distribution of inferior tibiofibular syndesmosis injuries repaired with screw fixation: a finite element study. PLoS One.

[34] Magerkurth O, Frigg A, Hintermann B, Dick W, Valderrabano V (2010). Frontal and lateral characteristics of the osseous configuration in chronic ankle instability. Br J Sports Med.

[35] Hintermann B, Boss A, Schafer D (2002). Arthroscopic findings in patients with chronic ankle instability. Am J Sports Med.

[36] Schafer D, Hintermann B (1996). Arthroscopic assessment of the chronic unstable ankle joint. Knee surgery, sports traumatology, arthroscopy : official journal of the ESSKA.

[37] Magerkurth O, Knupp M, Ledermann H, Hintermann B (2006). Evaluation of hindfoot dimensions: a radiological study. Foot & ankle international.

[38] Shah AS, Kadakia AR, Tan GJ, Karadsheh MS, Wolter TD, Sabb B (2012). Radiographic evaluation of the normal distal tibiofibular syndesmosis. Foot & ankle international.

[39] Evans SP, Parr WC, Clausen PD, Jones A, Wroe S (2012). Finite element analysis of a micromechanical model of bone and a new 3D approach to validation. J Biomech.

[40] Clanton TO, Viens NA, Campbell KJ, Laprade RF, Wijdicks CA (2014). Anterior talofibular ligament ruptures, part 2: biomechanical comparison of anterior talofibular ligament reconstruction using semitendinosus allografts with the intact ligament. Am J Sports Med.

